# Integration of artificial intelligence competencies into a holistic digital literacy framework for middle school education

**DOI:** 10.1038/s41598-026-47133-1

**Published:** 2026-04-17

**Authors:** Raghda Altamimi, Anas Abu Al-Haija’a

**Affiliations:** Institute of Computer Science, Faculty of Informatics of Eötvös, ELTE, Loránd University, Budapest, Hungary

**Keywords:** Artificial intelligence (AI), Artificial intelligence in education AIED, Digital literacy framework, Middle school education, Educational frameworks, Computational thinking, Digital citizenship, AI Ethics, AI Literacy, Mathematics and computing, Science, technology and society

## Abstract

The rise of artificial intelligence (AI) and its rapidly expanding tools is revolutionizing various sectors, with education standing out as a profoundly affected domain, creating an urgent need to incorporate AI competencies into education. This study proposes an AI-enriched digital Educational Framework (AIEDF) based on a newly developed Holistic Digital Literacy Framework (HDLF), synthesizing competencies from existing frameworks. It investigates the essential AI competencies for preparing young learners, specifically middle-school students, to prosper in an AI-driven world. Middle-school students are targeted due to their developmental readiness for abstract thinking, allowing effective early engagement with foundational AI concepts. Initially, four established digital education frameworks are analyzed and synthesized to create HDLF to be used as the foundation for incorporating AI competencies suitable for the cognitive level of the intended age group. The HDLF contains four core areas (Core Computational Concepts and Skills, Digital Citizenship and Ethical Competence, Impact of Computing on Society, and Digital Competence and Lifelong Learning). A literature review identified AI competencies essential for thriving in the 21st century. Using a systematic integration approach, these competencies were analyzed and mapped to the subtopics of the HDLF. The resulting AIEDF is then aligned with Bloom’s taxonomy, illustrating the progression from fundamental AI understanding (e.g., Machine Learning Basics) to more advanced tasks (e.g., designing simple AI applications). It is designed to support structured AI education, developmentally appropriate, and aligned with international standards, proposing a structured and adaptable foundation that requires empirical validation in future work.

## Introduction

AI refers to systems or machines capable of analyzing their environment, processing large amounts of data, and performing tasks like reasoning, learning, problem-solving, and decision-making (traditionally requiring human intelligence) with some degree of autonomy^1–3^. It is increasingly influencing various facets of modern life, including healthcare, transportation, finance, and education^4–7^, necessitating a paradigm shift in how learners engage with technology. As AI becomes ubiquitous, preparing future generations to critically navigate, innovate, and ethically shape an AI-driven world is imperative^8–10^. Middle school education serves as a pivotal stage for introducing these competencies, as students begin to develop abstract thinking and can grasp foundational concepts that underpin AI technologies^11^. As digital literacy continues to evolve in the age of AI, educational frameworks must adapt to incorporate new skill sets, emphasizing not only technical proficiency but also ethical considerations and digital citizenship^12^. Recent scoping work on generative AI in education, drawing on a review of 453 articles, has proposed a comprehensive taxonomy of applications and challenges and emphasizes the need for ethical considerations and informed policies, reinforcing calls for coherent, age-appropriate AI competency frameworks in schooling^13^. However, existing digital education frameworks often lack a cohesive integration of technical, ethical, and societal AI competencies, creating a gap in preparing students for holistic engagement with AI technologies^14,15^.

This study addresses this gap by developing an enriched informatics education framework that integrates essential AI competencies tailored for middle school students. Unlike prior efforts that focus narrowly on technical skills or theoretical ethics, this framework bridges computational proficiency with ethical reasoning, societal awareness, and lifelong learning. By embedding AI concepts across core digital domains, the framework aims to equip students to use, critique, and innovate AI technologies responsibly.

To establish a robust foundation for the underlying digital framework that will subsequently integrate AI competencies, four prominent digital education frameworks were analyzed:

1. DigComp 2.2: The Digital Competence Framework for Citizens emphasizes digital literacy and citizenship^16^;
2. K–12 Computer Science Framework prioritizes computational thinking and programming^17^;
3. Informatics Reference Framework for School (2022) focuses on foundational informatics^18^;
4. Informatics Reference Framework for School (2023) introduces emerging topics like AI, Virtual Reality (VR), and Augmented Reality (AR)^19^.

These frameworks were selected for their complementary strengths: Dig Comp’s holistic digital citizenship focus, K–12’s technical rigor, and the Informatics frameworks’ evolving emphasis on emerging technologies. A synthesis of these frameworks revealed critical gaps in addressing AI’s interdisciplinary nature, motivating the development of HDLF. It integrates computational, ethical, societal, and lifelong learning domains, providing a scaffolded structure for embedding AI competencies.

Through a competency-based mapping, AI concepts were mapped to the HDLF’s domains, ensuring alignment with middle schoolers’ cognitive development and educational standards. The resulting AIEDF balances technical mastery (e.g., data literacy), ethical deliberation (e.g., AI safety), and societal engagement (e.g., inclusive design). The AIEDF competencies are aligned with Bloom’s Taxonomy, scaffolding learning from foundational knowledge (Remember, Understand) to advanced innovation (Evaluate, Create). For instance, students progress from interpreting AI-driven data visualizations to designing inclusive AI tools for community challenges, fostering both technical mastery and ethical reasoning.

This work contributes to the field of AI education research by introducing a structured methodology for integrating AI into existing digital frameworks. thereby addressing the fragmentation observed in current approaches. It further presents a developmentally appropriate model that bridges both technical and socio-technical competencies, ensuring that learners receive a holistic education. Moreover, the framework offers a theoretical foundation to help educators conceptualize AI literacy integration without compromising core curricular objectives. By theoretically outlining how students can be equipped to both leverage and critically scrutinize AI technologies, this conceptual work advances the broader goal of cultivating informed, ethical participants.

This study follows a design-oriented framework development approach: we (i) synthesize HDLF from established digital education frameworks, then (ii) synthesize AI education competencies from the literature, and (iii) integrate the resulting AI competencies into HDLF to form AIEDF, followed by Bloom-aligned learning progressions. The work is “systematic” in the sense that each stage uses explicit units of analysis, decision rules, and traceable outputs (tables) that enable replication of the synthesis and mapping procedure.

- RQ1. How can domains/subdomains from selected international digital education frameworks be synthesized into a unified HDLF suitable for middle school education?
- RQ2. Which AI competency statements are most relevant in contemporary AI literacy / K–12 AI education literature as developmentally appropriate for middle school learners?
- RQ3. How can these AI competency domains be mapped onto HDLF subtopics and aligned with Bloom’s Taxonomy to construct a coherent AIEDF?

Following the introduction, the paper presents two analytical background sections. Existing Digital Education Frameworks synthesizes and compares selected digital education frameworks to identify convergent competency areas and gaps relevant to middle school contexts. AI Competencies in Middle School Education synthesizes AI literacy and K–12 AI education literature to specify AI competency domains appropriate for middle school learners, including technical, ethical, and societal dimensions. The Methodology section then details the staged qualitative synthesis and competency-mapping procedure used to construct the HDLF and integrate AI competencies into the AIEDF, including developmental alignment through Bloom’s Taxonomy. Subsequent methodology subsections present the resulting AIEDF and the explicit alignment of integrated competencies to support pedagogical progression. A Planned Empirical Validation subsection outlines the proposed staged validation pathway and implementation-oriented evaluation directions. The paper then delineates the limitations of the study, explicitly noting the absence of empirical validation and implementation testing, before concluding with the Conclusion and Future Work sections, which summarize findings and outline priorities for empirical evaluation and school-level operationalization.

### Existing digital education frameworks

As the first analytical output of the framework-synthesis procedure (Stage 1; see Sect. "Methodology"), this section reviews the selected digital education frameworks and synthesizes their coverage and gaps with respect to middle school digital literacy and emerging AI-related needs. The DigComp 2.2 framework provides a comprehensive model for digital citizenship, emphasizing information literacy, safety, and problem-solving. However, its AI competencies, such as understanding recommendation systems or automated decision-making, are generalized for adult citizens, lacking age-appropriate adaptations for middle school learners’ cognitive and ethical development^16^. Similarly, the K–12 Computer Science Framework^17^ advances computational thinking and programming but treats AI as a peripheral topic, with minimal guidance on machine learning, ethics, or real-world AI applications^8,17^. This omission is particularly critical for middle schoolers, who require structured scaffolding to transition from basic coding to AI-driven problem-solving^8^.

The Informatics Reference Frameworks (2022/2023)^18,19^ position AI as a standalone core topic within computational concepts (e.g., algorithms, programming) but fall short of integrating AI into broader digital competency domains such as digital citizenship, ethics, or societal impact. For instance, while AI is acknowledged in technical contexts (e.g., machine learning workflows), its ethical implications such as bias in automated decision-making or AI’s role in misinformation are siloed rather than woven into discussions of digital responsibility^20^. This compartmentalization limits students’ ability to critically evaluate AI’s societal consequences, a gap echoed in UNESCO’s critique of AI education’s “technical-first” focus^21^.

### AI competencies in middle school education

As the second analytical output of the methodological workflow (Stage 2; see Sect. "Methodology"), this section synthesizes the AI literacy literature to identify AI competency areas that are developmentally appropriate for middle school learners and relevant to international AI education directions. Integrating AI competencies at a young age not only fosters interest in STEM fields but also enhances the digital literacy essential for thriving in the 21st century^12,22^. Indeed, emerging research underscores the need for early AI education in K–12 curricula^23,24^. For example^8^ advocate for a structured approach to AI education, offering comprehensive guidelines on the AI knowledge every child should acquire. Furthermore, a collaborative initiative by the International Society for Technology in Education (ISTE) and the Computer Science Teachers Association (CSTA) has yielded a set of AI education standards that delineate core concepts and best practices for students^25^. These standards, encapsulated in the Big 5 ideas, encompass critical AI topics such as Perception, Representation and Reasoning, Machine Learning, Human-AI Interaction, and AI’s Societal Impact.

Research indicates that early exposure to AI concepts enhances students’ analytical skills, computational thinking, and ethical reasoning^26–28^. Recent research highlights the importance of integrating AI ethics into early education, ensuring that students not only acquire technical competencies but also develop the critical ethical reasoning needed to navigate the challenges posed by emerging technologies^29^. Studies show that integrating AI into middle school curricula can increase student engagement and understanding of complex technological systems^30–32^. Furthermore, ethical education in AI is crucial for developing responsible digital citizens who are aware of the societal and ethical implications of technology^33–35^.

Despite the recognized importance, several challenges hinder the integration of AI competencies into middle school education. These include a lack of teacher preparedness, limited curricular resources, and the abstract nature of AI concepts^8,36,37^. Addressing these challenges requires the development of comprehensive teacher training programs, accessible educational materials, and age-appropriate instructional strategies. Fostering an inclusive learning environment through differentiated instruction and culturally responsive teaching can promote equity and diversity within AI education.

The AIEDF addresses fragmentation in AI education by unifying technical, ethical, and societal competencies, while its modular design enables future extensions to tackle unresolved challenges such as teacher preparedness and equitable resource access. For instance, the framework’s scaffolded structure supports the integration of teacher training modules and low-cost AI tools adaptations planned for subsequent phases of implementation. This adaptability ensures the AIEDF can evolve alongside technological and pedagogical advancements, fostering equitable, future-ready AI literacy.

## Methodology

### Research design

This study employs a conceptual framework development research design^38^. It does not report empirical outcomes; instead, it develops and specifies two conceptual artifacts: (i) HDLF derived through structured synthesis of established digital education frameworks, and (ii) an AIEDF created by integrating AI competency domains into HDLF and aligning them with Bloom’s Taxonomy. This aligns with established guidance on qualitative content analysis as a stepwise, rule-guided approach and on conceptual frameworks as a method for organizing and systematizing knowledge.

**Figure 1:**
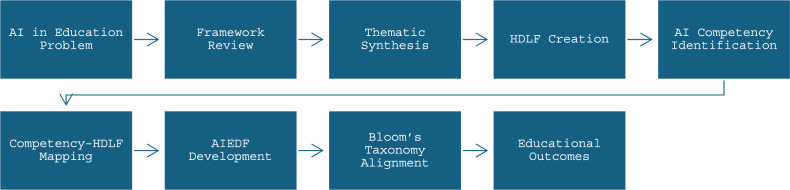
Staged methodological workflow for HDLF synthesis and AIEDF construction.

Stage 1 conducts framework synthesis by segmenting competency statements from the selected reference frameworks into statement-level units, coding them using a documented codebook, and integrating them into HDLF domains and subdomains through traceable synthesis tables. Stage 2 conducts AI competency synthesis by extracting and consolidating AI competency statements from the AI education literature into the core competency clusters used in this study. Stage 3 applies explicit competency-based mapping rules to map atomic AI competency statements to HDLF subdomains (including concept dependence, learning-objective alignment, and socio-technical locus), with primary/secondary assignment for cross-cutting competencies, to construct the AIEDF. Bloom’s Taxonomy is then used to translate the resulting competencies into measurable learning objectives and assessment-oriented learning progressions. Reproducibility is supported through traceable outputs (tables), coder consensus procedures, and explicit coding and mapping rules; empirical validation (e.g., expert review and classroom pilots) is positioned as subsequent work. The overall workflow is illustrated in Fig. 1.

### The development of the HDLF

To establish a comprehensive foundation for the AIEDF, four prominent digital education frameworks were selected based on their relevance, comprehensiveness, and influence on educational policy, research in digital education, and curriculum development. These frameworks (DigComp 2.2^16^, K–12 Computer Science Framework^17^, and Informatics Reference Frameworks (2022/2023)^18,19^) were chosen for their complementary strengths in digital citizenship (DigComp 2.2), technical rigor (K–12 CS), and emerging AI focus (Informatics). Table 1 summarizes why these four frameworks were chosen, highlighting their distinctive strengths and the specific gaps our AIEDF aims to address. By examining their complementary focuses (digital citizenship, technical rigor, and emerging AI considerations), we create a more holistic foundation suited for middle school learners (Table 2).

**Table 1:** Framework selection rationale for HDLF development.

Framework	Strengths	Gaps addressed by AIEDF
DigComp 2.2	Digital citizenship, lifelong learning	Lacks AI technical skills (e.g., neural networks) and age-appropriate AI technical depth
K–12 CS Framework	Programming, algorithms, computational thinking	Omits AI ethics (e.g., bias mitigation) and societal impact (e.g., AI-driven equity issues)
Informatics 2022/2023	AI ethics, sustainability, and applied problem-solving	Limited interdisciplinary linkages (e.g., AI’s role in misinformation)

**Table 2:** Selection and exclusion criteria for reference framework selection.

Criterion type	Criterion	Operational definition (how judged)	Example(s) of application
Inclusion	Public + stable	Official release/version, accessible documentation	DigComp 2.2^16^, K–12 CS Framewor^17^, Informatics Reference Framework for School (2022) and (2023) ^18,19^
Inclusion	Educational framework	Intended to guide curriculum/competencies (not a local syllabus)	DigComp 2.2^16^, K–12 CS Framework^17^, Informatics Reference Framework for School (2022) and (2023)^18,19^
Inclusion	Granularity	Domains/subdomains + competency statements enabling coding	DigComp 2.2^16^, K–12 CS Framework^17^, Informatics Reference Framework for School (2022) and (2023)^18,19^
Inclusion	Middle-school suitability	K–12 focus or clearly scaffoldable for Grades 6–8	K–12 CS Framework^17^, Informatics 2022 and 2023^18,19^ (target audience = K–12 students); DigComp 2.2^16^ is included as scaffoldable but noted as adult-oriented in parts
Inclusion	Influence	Evidence of adoption/ endorsement and/or strong uptake in policy/ curriculum/ research practice	DigComp 2.2^16^ (EU/JRC framework widely used in policy and practice); K–12 CS Framework^17^ (widely referenced in K–12 curriculum design and standards alignment); Informatics Reference Framework for School (2022) and (2023)^18,19^ (used as an emerging informatics/AI-oriented school reference framework)
Exclusion	Redundancy	High overlap with selected frameworks without added structure	ISTE Standards for Students^41^; Common Sense Digital Citizenship^42^; excluded when domains are already covered by the four selected frameworks and no distinct coding-relevant structure is added
Exclusion	Too narrow	Covers only one domain	Exclude single-domain frameworks (e.g., media literacy only^43^) if scope is broader digital/AI competencies
Exclusion	Insufficient detail	Too little competency detail for reproducible coding	OECD AI Principles^44^; UNESCO Recommendation on the Ethics of Artificial Intelligence^41^ch(normative/policy principles, not domain/subdomain competency statements for reproducible coding)
Exclusion	Unstable/unclear provenance	No identifiable version/release or incomplete documentation	No fixed named framework was pre-specified for exclusion under this criterion; rather, the criterion was applied case-by-case to documents (e.g., webpages, slide decks, draft frameworks) lacking a clearly identifiable issuing body, version/release information, and/or complete documentation

### Framework selection and exclusion rationale

To strengthen transparency and generalizability, the selection of reference frameworks followed a documented scoping-and-screening procedure. First, we defined a priori purpose statement: the HDLF required (i) a comprehensive digital competence component (citizenship, safety, communication, lifelong learning), (ii) a K–12-appropriate computing education component (computational thinking, programming, algorithms, data), and (iii) an informatics-oriented perspective that explicitly addresses emerging technologies (including AI) and the relationship between technical competencies and broader societal/ethical implications. Second, we applied explicit inclusion criteria: frameworks had to be (1) publicly available and stable (official documentation with identifiable version/release), (2) intended for education/curriculum guidance or competency definition (not a single-institution syllabus), (3) sufficiently granular to support competency extraction and mapping (domains/subdomains, competency descriptions, or learning outcomes), and (4) applicable to, or adaptable for, the middle-school level (either explicitly K–12 or readily scaffoldable). Third, we operationalized “influence” as a composite indicator based on: (a) formal adoption or endorsement by recognized organizations or jurisdictions (e.g., governmental/standards bodies), and/or (b) demonstrable uptake in education policy, curriculum development, or widely cited research/practice guidance.

Frameworks were excluded when they (i) substantially overlapped with selected frameworks without adding new competency structure needed for synthesis, (ii) were highly regional without transferable competency descriptions (or lacked sufficient documentation in a stable form), (iii) were narrow to a single domain (e.g., only technical computing, or only media literacy) and therefore could not support the HDLF’s intended holistic scope, or (iv) did not provide enough competency granularity to enable transparent thematic coding and mapping.

Finally, we acknowledge and mitigate potential regional and language bias. Two of the selected references originate from European contexts (DigComp 2.2 and the Informatics reference frameworks^16,18,38^), which may reflect European policy priorities and terminology. To reduce this risk, the K–12 Computer Science Framework^17^ serves as a complementary anchor that is widely used beyond Europe^39,40^, and more importantly, the synthesis is done at the level of transferable competencies (e.g., data literacy, safety, ethics, societal impact) rather than local policy language. HDLF is explicitly positioned as a modular, adaptable structure intended for localization. Global applicability is included as a limitation and future validation target (e.g., expert review and pilot alignment in non-European settings).

A qualitative content analysis was conducted to extract core components and competencies from each framework. The consolidation process involved a systematic three-phase approach to analyze and synthesize the selected frameworks. First, thematic coding was conducted through iterative researcher discussions, identifying core themes (e.g., computational thinking, digital ethics, societal impact) across frameworks. Second, a comparative synthesis highlighted overlaps such as shared emphases on data literacy and programming while exposing critical gaps, including DigComp 2.2’s limited integration of AI-specific technical skills (e.g., neural networks) scaffolded for middle schoolers, the K–12 CS Framework’s complete omission of AI concepts, and the Informatics Frameworks introduce AI topics (e.g., machine learning) but isolate technical skills from digital citizenship (e.g., no guidance on evaluating AI’s role in cyberbullying). Finally, a holistic integration synthesized competencies into a unified structure, prioritizing middle school developmental stages, ensuring pedagogical actionability. Tables 2 and 3 provide a closer examination of the selected frameworks. Table 2 offers a thematic comparison, outlining their primary focus, target audience, problem-solving approach, and identified gaps, and Table 3 highlights the core competency domains covered by each framework.

**Table 3:** Thematic comparison of selected reference frameworks.

Theme	DigComp 2.2	K–12 CS framework	Informatics 2022	Informatics 2023
Primary Focus	Digital citizenship	Technical CS education	Foundational informatics	Advanced informatics + ethics
Problem-Solving Approach	Practical tool usage	Algorithmic thinking	Applied modeling	AI-driven solutions
Target Audience	All citizens	K–12 students	K–12 students	K–12 students
Skill Progression	Lifelong learning	Grade-level scaffolding	Conceptual scaffolding	Conceptual + emerging tech
Gaps	AI content is adult-oriented and lacks scaffolded instruction in AI ethics for middle school learners	Excludes AI entirely, omitting integration of ethical and societal considerations	Does not address AI competencies, leaving a gap in technical AI skills	Integrates advanced informatics and ethics but falls short of fully linking technical AI competencies with societal impact

The unit of analysis was an individual competency statement/descriptor extracted from each reference framework (i.e., domain/subdomain definitions and competency examples were segmented into statement-level units). Extracted units were compiled into a comparison matrix (framework × statement). A codebook (v1) was developed using a hybrid approach: (i) deductive codes based on recurring competency areas across the four frameworks (e.g., data literacy, programming/algorithms, safety, ethics, societal impact, collaboration), and (ii) inductive refinement when framework-specific concepts did not fit existing codes. Coding was conducted by two coders (Author 1 and Author 2). When more than one coder was involved, disagreements were resolved through brief consensus discussions using explicit inclusion/exclusion rules in the codebook, and the codebook was updated to a stable version (v2). A short codebook excerpt is provided in Table 4 to illustrate coding boundaries and support reproducibility Table 5.

**Table 4:** Codebook excerpt illustrating thematic codes, inclusion/exclusion rules, and roundaries.

Code (theme)	Definition	Include when…	Exclude when…	Example (statement-level unit)
Data literacy/analysis	Skills for collecting, organizing, interpreting, and visualizing data	Focus is on data handling, interpretation, and visualization	Focus is purely on ethics/safety without data practices	“Collect and interpret data to identify patterns…”
Ethics & responsibility	Normative judgment, fairness, accountability, and responsible use	Explicit ethical reasoning, fairness/bias, accountability	Only a technical description without an ethical dimension	“Evaluate fairness/bias in automated decisions…”
Societal impact	Broader effects on people/society (equity, misinformation, labor, culture)	Mentions societal consequences, inclusion/equity, and misinformation	Individual safety/privacy only	“Analyze how technology shapes opinions or equity…”

**Table 5:** Comparative coverage of competency domains across selected frameworks. ✓▲ denotes partial coverage.

Competency domain	DigComp 2.2	K–12 CS framework	Informatics 2022	Informatics 2023
Information and Data Literacy	✓	✓	✓	✓
Programming	✓	✓	✓	✓
Digital Communication	✓	×	✓	×
Content Creation	✓	×	×	×
Digital Safety	✓	×	✓	×
Ethics & Societal Impact	✓	✓	✓▲	✓
Lifelong Learning	✓	×	×	×

A synthesis of recurring and unique competencies across established frameworks reveals distinct yet overlapping domains of digital expertise. The DigComp 2.2 emphasizes information literacy such as evaluating sources and managing data alongside digital communication and collaboration, content creation with an awareness of multimedia production and copyright considerations, digital safety that covers aspects of privacy and cybersecurity, and the use of digital tools for problem-solving^16^. In contrast, the K–12 CS Framework^17^ centers on computational thinking, which includes abstraction, algorithmic design, decomposition, programming skills, and data analysis and visualization, but does not address AI in any capacity a gap addressed by the AIEDF’s explicit integration of AI competencies.

Complementing these, recent informatics frameworks (2022/2023) designed for school settings integrate foundational informatics concepts such as Algorithms, Data and Information, and AI, explicitly addressing AI’s technical dimensions (e.g., machine learning, robotics) and ethical considerations^18,19^. However, they do not systematically connect AI competencies to broader digital citizenship domains such as safety, equity, or societal impact, a gap the AIEDF resolves by embedding AI ethics and societal evaluation across all core areas. For instance, while the Informatics frameworks introduce AI topics like machine learning, they lack structured guidance for middle schoolers to critically evaluate AI’s societal risks (e.g., misinformation, privacy erosion) or design inclusive AI systems, competencies central to the AIEDF’s Digital Citizenship and Societal Impact domains.

Digital education frameworks exhibit critical gaps, primarily a disconnect between technical AI competencies and their societal implications. Existing models address these aspects in isolation, failing to integrate technical skills such as neural network programming with ethical considerations like algorithmic bias. For instance, DigComp 2.2 introduces AI literacy through concepts such as recommendation systems, yet its content is designed for adult learners and does not account for middle school students’ cognitive development, including the need for scaffolded instruction in AI ethics and model training. Similarly, while the K–12 CS Framework emphasizes algorithmic thinking, it excludes AI, whereas Informatics frameworks focus on technical AI applications (e.g., machine learning workflows) but neglect digital citizenship components such as online collaboration. Addressing these gaps requires the integration of DigComp’s comprehensive approach to digital literacy, including safety and ethics, with the technical rigor of the K–12 CS and Informatics frameworks. A unified model could, for example, engage middle school students in designing a recommendation algorithm while critically analyzing its potential biases in disseminating misinformation, thus fostering both technical proficiency and ethical awareness.

#### Criteria for integration

The consolidation process aimed to develop a unified framework that integrates the strengths of each framework while addressing existing gaps. To achieve this, four evidence-based integration criteria were established:

1. Adaptability to Middle School Cognitive Levels: competencies were prioritized to match middle school students’ cognitive and developmental stages, ensuring appropriateness and engagement with complex concepts. For instance, programming concepts are introduced through block-based platforms like Scratch to simplify abstract logic.
2. Comprehensiveness: The framework was designed to merge technical rigor (K–12 CS) with sociotechnical domains (DigComp’s digital citizenship) and encompass all domains necessary for preparing students to navigate the digital world effectively, ensuring no essential areas were overlooked.
3. Clarity and Usability: The framework was designed to be understandable and implementable, facilitating seamless integration into educational curricula.
4. Alignment with Educational Standards: The framework was aligned with international benchmarks (e.g., DigComp 2.2’s digital citizenship^16^, K–12 CS’s computational thinking^17^) to ensure pedagogical relevance.

By adhering to these criteria, **four core domains** were identified as the HDLF’s foundation:

1. Core Computational Concepts and Skills

This domain integrates foundational technical competencies such as programming, algorithms, data analysis, and computational thinking. These are essential for students to understand and engage with technology effectively. The HDLF is adaptable to different cognitive levels by using age-appropriate examples and hands-on activities to simplify complex computational concepts. For example, middle school students might engage in interactive programming exercises using block-based coding platforms such as Scratch, work with robotics kits like LEGO Mindstorms to solve tangible problems, and collaborate on data collecting, analyzing, and visualizing projects such as the school’s daily electricity usage, thereby reinforcing core computational concepts in an age-appropriate, hands-on manner. These comprehensive core competencies underpin digital literacy and support robust learning in computer science and AI^45–48^. Additionally, by emphasizing clear approaches to programming and algorithms, the HDLF ensures it is understandable and usable, facilitating seamless integration. Finally, by aligning with frameworks such as DigComp 2.2 and the K-12 Computer Science Framework^16,17^, this domain meets established benchmarks for foundational digital education skills.

2. Digital Citizenship and Ethical Competence

This domain focuses on ethics, responsibility, safety, and well-being. As students increasingly interact within digital environments, it is crucial for them to understand responsible use, ethics, and online safety^49–51^. Education in digital citizenship plays a significant role in reducing instances of cyberbullying and promoting positive online behavior^52–55^. By instilling a sense of ethical responsibility and awareness of safe practices, students are better equipped to navigate digital spaces responsibly, fostering a respectful and secure online community^53,55,56^. Moreover, by comprehensively addressing technical skills and ethical understanding, this domain equips learners with a holistic digital identity that values the human dimension of technology use The clarity and usability of this domain are strengthened by clearly defining this core area, enabling seamless integration of digital responsibility into various subjects. For example, a curriculum might include a dedicated module on digital citizenship where students engage in role-playing exercises to practice responsible online behavior, analyze real-world case studies of ethical dilemmas in digital communication, and collaborate on projects that simulate decision-making processes in cybersecurity scenarios. Furthermore, emphasizing digital citizenship aligns with global and national standards that advocate for fostering responsible and safe online engagement as an essential component of digital competence^57–60^.

3. Impact of Computing on Society

This domain examines the broader social, cultural, and economic implications of technology. It connects complex societal issues to students by relating them to students’ everyday experiences, such as how social media shapes opinions, making this accessible and meaningful^61–63^. By addressing both the positive impacts and ethical challenges of technology, it fosters critical thinking and informed decision-making, helping students understand that technology is not neutral and can affect equity, inclusion, and ethical choices^64–66^. Additionally, encouraging the critical evaluation of technological changes aligns with educational frameworks that promote informed citizenship and the responsible use of digital tools^67^.

4. Digital Competence and Lifelong Learning

This domain emphasizes cultivating a mindset of continuous skill development. It introduces growth, adaptability, and ongoing learning in an age-appropriate manner, encouraging middle school students to stay curious and open to technological advances. Developing continuous learning skills is essential for adapting to rapid technological changes, with lifelong learning competencies linked to better employment outcomes and personal growth^25,26,68,69^. By highlighting the importance of ongoing skill development, the framework ensures that students are prepared for current technologies and equipped to adapt to new ones, maintaining long-term relevance. The domain enhances clarity and usability by guiding learning strategies such as self-assessment, goal setting, and leveraging online resources. Additionally, emphasizing adaptability and digital skill development aligns with educational standards, supporting recognized learning outcomes and enabling widespread adoption, thus preparing students for a rapidly evolving digital economy and society^70,71^.

These core areas incorporate essential digital competencies, laying the groundwork for AI integration in the next step. Building on these analyses, Table 6 delineates the HDLF’s core areas, subtopics, and reference frameworks. This table shows how we integrated elements from each existing framework into a comprehensive structure designed to guide AI-enriched digital literacy.

**Table 6:** HDLF core areas, subtopics, descriptions, and source reference frameworks.

Core Area	Subtopic	Description	Ref FW
**Core Computational Concepts and Skills**	Data Science and Analysis	Develops data literacy through structured/unstructured data handling. Students collect (surveys), organize (spreadsheets), and analyze data (mean, frequency) to identify patterns. Visualization (graphs/heatmaps) via digital tools enhances analytical reasoning. Supported activities integrate ethics (privacy, bias) and real-world applications (environmental trends), enabling predictive modeling and insight communication	^16–19^
	Programming and Algorithms	Develops computational thinking via algorithm design using block-based (Scratch) and text-based (Python) programming. Students learn core concepts (variables, control structures) through contextual tasks. Modular decomposition and iterative debugging foster systematic reasoning and resilience. Integrates procedural and object-oriented principles with real-world applications	^17–19^
	Computing Systems	Explores hardware-software interdependencies (e.g., CPUs, memory, IoT devices) via supported activities, analogies (e.g., nervous system), and troubleshooting (e.g., resolving software malfunctions). Students simulate system limitations (data overload) and engage in collaborative projects (assembling computers) to grasp computational logic	^17,18^
**Digital Citizenship and Ethical Competence**	Ethics and Responsibility	Engages students in individual ethical decision-making through relatable, age-appropriate dilemmas (e.g., data-sharing choices in gaming communities, plagiarism in digital projects). Role-playing (e.g., responding to cyberbullying) and harm/benefit frameworks foster awareness of personal accountability in digital interactions	^16–19^
	Safety and Well-being	Cultivates technical safeguards (e.g., password hygiene, malware detection tools) and behavioral strategies (e.g., screen-time journals, empathy-driven conflict resolution) through experiential learning. Simulations like “Phishing Attack Labs” and peer-designed “Digital Wellness Campaigns” (e.g., promoting ergonomic practices) bridge abstract risks to tangible habits. Metaphors like “mental firewalls” (emotional boundaries) and “digital immune systems” (device security) align with concrete operational thinking, while integration with CASEL’s social-emotional learning (SEL) standards reinforces holistic wellness ^72^	^16–19^
**Impact of Computing on Society**	Equity and Inclusion	Addresses systemic barriers in technology by fostering inclusive design. Students analyze exclusion in digital tools, create accessible solutions like tactile programming interfaces, and engage in culturally relevant projects, such as IoT-based Indigenous land monitoring. Competency-based assessments, including WCAG compliance, ensure accessibility and equity in computing education	^16–19^
	Societal Impact	Examines macro-level consequences of computing, such as global e-waste flows, and gig economy labor practices. Students engage in systems thinking via simulations (e.g., Minecraft: Education Edition sustainable city designs) and debates on policies like “Should schools allow smartwatches in classrooms?”. Civic projects, such as mapping local air quality with Arduino sensors or analyzing viral misinformation’s societal ripple effects, foster global citizenship aligned with ISTE’s Global Collaborator standards ^73^	^16–19^
**Digital Competence and Lifelong Learning**	Communication and Collaboration	Develops students’ capacity for ethical, effective digital engagement through collaborative problem-solving (e.g., coding shared projects), netiquette (role-playing conflict resolution), and critical analysis of social media biases. Guided pedagogy integrates secure communication practices (encrypted chats, phishing awareness) and network literacy analogies (e.g., “data packets as postcards”), aligning with ISTE and CSTA standards to foster responsible digital citizenship ^73^	^16–19^
	Problem Solving with Technology	This subtopic develops structured problem-solving skills through technical challenges (e.g., debugging code via iterative design cycles) and creative applications (e.g., repurposing spreadsheets for algorithmic art). Guided activities, from tactile robotics to collaborative hackathons, foster systematic troubleshooting, empathy-driven innovation (e.g., community apps), and metacognitive growth, bridging concrete operational tasks to abstract reasoning (e.g., smart city simulations)	^16–18^
	Content Creation	This subtopic equips students to design, iterate, and ethically share digital artifacts (e.g., interactive stories, simulations) using tools like Scratch and Python. Projects integrate user-centered design, remixing media for inclusivity, and intellectual property literacy. Scaffolded progression from remixing templates to coding simulations fosters computational fluency and systems thinking, aligning with ISTE and CSTA standards for creative, ethical communication ^73^	^16–19^

A qualitative content analysis was conducted to extract core components and competencies from each framework. The consolidation process involved a systematic three-phase approach to analyze and synthesize the selected frameworks. First, thematic coding was conducted through iterative researcher discussions, identifying core themes (e.g., computational thinking, digital ethics, societal impact) across frameworks. Second, a comparative synthesis highlighted overlaps such as shared emphases on data literacy and programming while exposing critical gaps, including DigComp 2.2’s limited integration of AI-specific technical skills (e.g., neural networks) scaffolded for middle schoolers, the K–12 CS Framework’s complete omission of AI concepts, and the Informatics Frameworks introduce AI topics (e.g., machine learning) but isolate technical skills from digital citizenship (e.g., no guidance on evaluating AI’s role in cyberbullying). Finally, a holistic integration synthesized competencies into a unified structure, prioritizing middle school developmental stages, ensuring pedagogical actionability. Tables 2 and 3 provide a closer examination of the selected frameworks. Table 2 offers a thematic comparison, outlining their primary focus, target audience, problem-solving approach, and identified gaps, and Table 3 highlights the core competency domains covered by each framework.

### The development of the AIEDF

AI competency domains were derived through a synthesis of AI literacy and K–12 AI education literature, focusing on sources that explicitly articulate learnable competencies or competency dimensions relevant to school-aged learners. Competency statements were extracted at the level of knowledge/skill/disposition, then consolidated by grouping overlapping concepts and refining domain labels for clarity and non-redundancy.

In addition to competencies explicitly articulated in AI literacy and K–12 AI education literature, cross-cutting meta-competencies (e.g., adaptability, collaboration, creativity) were retained when they were (a) repeatedly associated with effective AI engagement in K–12 contexts or (b) embedded within competency-model formulations of AI literacy. Representative competency statements were mapped to each domain to preserve traceability between the synthesized literature and the resulting framework. The resulting competency domains are presented in The Development of the AIEDF subsection and operationalized through the mapping in Table 7, with the final integrated AIEDF shown in Table 8.

1. Fundamental AI Knowledge and Understanding

**Table 7:** Mapping of literature-derived AI competencies to HDLF domains and subtopics.

AI competency	Aligned digitalframework Areas	Rationale
**1. Fundamental AI Knowledge and Understanding**	- **Core Computational Concepts and Skills:**	AI’s foundational principles (e.g., machine learning, neural networks) require a strong grounding in programming logic, algorithmic design, and data interpretation key skills within these subtopics
	• Programming and Algorithms	
	• Computing Systems	
	• Data Science and Analysis	
**2. Data Literacy and Computational Thinking**	- **Core Computational Concepts and Skills:**	Mastery of data collection, cleaning, analysis, and visualization directly corresponds with the digital framework’s emphasis on data science, while the algorithmic reasoning aspect reinforces computational thinking skills
	• Data Science and Analysis	
**3. Practical Application and Operational Competence**	- **Core Computational Concepts and Skills:**	Hands-on AI application requires coding, debugging, and deploying AI models. Learners should also develop skills in AI model optimization, improving performance in machine learning workflows (e.g., hyperparameter tuning, neural network optimization)
	• Programming and Algorithms	
	- **Digital Competence and Lifelong Learning:**	
	• Problem Solving with Technology	
	• Content Creation	
**4. Critical Evaluation, Creativity, and Innovation**	- **Impact of Computing on Society:**	Evaluating AI outputs and innovatively repurposing AI technology involve assessing societal and ethical implications. This includes AI-driven creativity, where learners use generative AI tools for content creation (text, images, and code). Additionally, learners should be able to leverage AI to enhance creative production and problem-solving
	• Societal Impact	
	- **Digital Competence and Lifelong Learning:**	
	• Problem Solving with Technology	
	• Content Creation	
	- **Digital Citizenship and Ethical Competence:**	
	• Ethics and Responsibility	
**5. Interdisciplinary Collaboration and Communication**	- **Digital Competence and Lifelong Learning:**	Effective AI integration requires the ability to work across disciplines and clearly communicate technical ideas, aligning directly with the framework’s focus on collaborative and communicative digital practices
	• Communication and Collaboration	
**6. Adaptability and Lifelong Learning**	- **Digital Competence and Lifelong Learning:**	The rapidly evolving nature of AI technologies necessitates continuous learning and flexible problem-solving strategies, which are central to the framework’s emphasis on lifelong learning and collaborative digital competence
	• Problem Solving with Technology	
	• Communication and Collaboration	
**7. Ethical, Legal, and Societal Implications**	- **Digital Citizenship and Ethical Competence:**	This competency ensures that learners develop a deep understanding of AI’s ethical dimensions data privacy, fairness, transparency, and accountability and its broader societal impact. It reinforces the framework’s commitment to ethical digital behavior and responsible innovation
	• Ethics and Responsibility	
	- **Impact of Computing on Society:**	
	• Societal Impact (and Equity and Inclusion)

**Table 8:** Representative crosscutting AI competencies and their multi-domain alignment from HDLF to AIEDF.

AI competency (literature-derived)	Mapped HDLF domain(s)	Conceptual fit
**1. Fundamental AI Knowledge and Understanding**	Core Computational Concepts and Skills (Programming and Algorithms; Computing Systems; Data Science and Analysis)	This competency aligns with **Core Computational Concepts and Skills** because foundational AI literacy requires learners to recognize AI systems, understand core AI technologies (e.g., machine learning, natural language processing, robotics, and IoT), and interpret AI representations and decision processes. These capabilities depend on computational reasoning grounded in **programming and algorithms**, **computing systems**, and **data-driven analysis**, which collectively provide the conceptual basis necessary for understanding how AI systems function and support the development of more advanced AI skills
**4. Critical Evaluation, Creativity, and Innovation**	Impact of Computing on Society (Societal Impact); Digital Competence and Lifelong Learning (Problem Solving with Technology, Content Creation); Digital Citizenship and Ethical Competence(Ethics and Responsibility)	This competency aligns with Impact of Computing on Society, Digital Competence and Lifelong Learning, and Digital Citizenship and Ethical Competence because critically evaluating AI outputs requires understanding their societal consequences, potential biases, and limitations. Creative and innovative uses of AI involve problem-solving, content generation, and exploratory design practices that correspond to lifelong learning and technological application. At the same time, responsible and ethical innovation requires judgment regarding fairness, transparency, and appropriate AI use, linking the competency to ethical and citizenship-oriented dimensions of the HDLF

In the field of AI education, learners must acquire a robust foundational knowledge of artificial intelligence, encompassing core topics such as machine learning, natural language processing, robotics, and the Internet of Things^74^. This foundational understanding not only demystifies complex AI technologies but also establishes a shared vocabulary that enhances interdisciplinary collaboration. Moreover, any comprehensive AI literacy framework must commence with a clear, conceptually grounded grasp of AI technologies as a prerequisite for the development of advanced skills^9^. Additionally, research such as “What is AI Literacy? Competencies and Design Considerations” further delineates critical competencies in this domain, including the ability to recognize AI, understand intelligence, differentiate between general and narrow AI, and evaluate representations, decision-making processes, and responses, thereby enriching the framework for effective AI education^75^.

2. Data Literacy and Computational Thinking

Since AI systems are fundamentally driven by data, learners must develop robust data literacy skills^17–19^, which include data collection, cleaning, analysis, visualization, and interpretation, and integrate these with computational thinking to understand algorithmic processes, cultivate coding proficiency, solve complex problems, and modify existing models as necessary. This integration of technical abilities, particularly algorithmic reasoning and coding, with a strong foundation in data literacy, is essential for learners to engage with AI systems at both conceptual and practical levels^9^. Moreover, “What is AI Literacy? Competencies and Design Considerations” research delineates critical competencies within this domain encompassing sensors, data literacy, learning from data, and critically interpreting data, thereby enriching and expanding the framework for effective AI education^75^.

3. Practical Application and Operational Competence

Competence in AI is not only theoretical. Learners must be able to apply, build, and optimize AI tools in real-world contexts^75^. This includes developing simple AI applications, using and fine-tuning pre-trained models, and designing AI algorithms to solve discipline-specific problems. Hands-on proficiency in AI model development and optimization bridges the gap between classroom learning and practical, relevant skills. Equally important is an understanding of the human role in AI, recognizing that humans play an important role in programming, choosing models, and fine-tuning AI systems^75^. The MAILS study demonstrates that effective AI literacy includes the “use and apply” dimension, emphasizing skills related to the practical deployment of AI technologies and fostering self-efficacy in problem solving^76^.

4. Critical Evaluation, Creativity, and Innovation

Beyond operating AI systems, it is crucial that learners can critically evaluate AI outputs and recognize potential biases, errors, or limitations, notably including the identification of AI hallucinations. This competency includes the ability to think creatively about how to enhance AI applications, driving innovation. Additionally, learners should explore AI-driven creativity, leveraging generative AI tools for content creation in text, images, and code, which requires developing foundational prompt literacy^8,31^. Furthermore, understanding AI’s strengths and weaknesses is essential, as it enables individuals to leverage the complementary capabilities of both AI and human expertise for more effective problem-solving^75^. Such skills ensure that users do not simply become passive consumers of AI output but active contributors to its evolution. Several studies emphasize that critical thinking and creative problem-solving are integral to AI literacy^9^. For example, the framework discussed by Faruqe et al. and corroborated by the broader literature on 21st-century skills (which highlight creativity and critical evaluation) call for these higher-order thinking skills as essential components of an effective AI education program^9^. Creativity and innovation were grouped with critical evaluation because multiple AI literacy frameworks position higher-order judgment, ideation, and design as interdependent competencies required for meaningful AI use^75,76^.

5. Interdisciplinary Collaboration and Communication

Effective AI integration into education requires learners to develop strong communication and collaboration skills, enabling them to work in interdisciplinary teams, explain complex AI concepts to non-specialists, and apply AI insights across various domains, from science to the humanities^31,34,77^. This competency is essential for translating technical knowledge into real-world solutions. AI’s role in education is expanding beyond STEM subjects, with applications like assistive technologies (e.g., text-to-speech tools) supporting special educational needs and disability (SEND) students^78^. To foster a holistic understanding of real-world challenges, designing an interdisciplinary framework that embeds AI across different areas, modeling effective teamwork, and ensuring AI’s relevance spans multiple areas is essential. This approach emphasizes project-based collaboration, where learners synthesize technical and non-technical perspectives and develop the skills to articulate, debate, and present AI-related ideas. By contextualizing AI within diverse disciplines and prioritizing inclusive communication, students are prepared for a future where AI transcends traditional academic boundaries, enhancing both digital literacy and essential interpersonal skills.

6. Adaptability and Lifelong Learning

Given the rapid pace of innovation in AI, a competency in adaptability is essential. Learners must cultivate a mindset geared toward continuous learning and be prepared to update their skills as technologies evolve. This includes the ability to critically assess emerging tools and trends and to change course when necessary. Faruqe et al. note that an effective AI competency framework must include provisions for lifelong learning, ensuring that individuals remain current in an ever-changing technological landscape^9^. This competency is also echoed in research on the future of work and digital transformation, where adaptability is recognized as a key driver of long-term success^69,79^.

7. Ethical, Legal, and Societal Implications

As AI becomes more pervasive, understanding its ethical dimensions is imperative^80^. This competency covers knowledge of data privacy, fairness, transparency, accountability, and the societal impacts of AI deployment. Learners must be prepared to address the moral dilemmas posed by AI, ensuring that technology is used responsibly and equitably. In addition, it is crucial to recognize that AI also risks propagating misinformation and exposing cybersecurity vulnerabilities^6,7,35^. Research in AI literacy consistently integrates ethics as a core pillar^8,28,34^. Both conceptual reviews and empirical studies (such as those summarized in the MAILS^76^) highlight the need for users to critically assess AI systems from an ethical standpoint, thereby embedding ethical reasoning within the fabric of AI competency^28,33–35^.

### HDLF and AIEDF competency mapping procedure and rules

To ensure methodological transparency and reproducibility, the mapping of AI competencies to the HDLF followed three explicit principles. First, conceptual alignment was applied: each AI competency was mapped to all HDLF domains whose learning objectives and conceptual scope directly corresponded to the competency’s core meaning. Second, multi-domain representation was permitted for inherently cross-cutting competencies (e.g., Critical Evaluation, Creativity, and Innovation), allowing a single competency to be associated with multiple HDLF domains when justified by shared pedagogical intent rather than forcing artificial exclusivity. Third, post-mapping refinement was conducted to enhance clarity and usability within the final AIEDF structure. This refinement included renaming competencies for pedagogical readability, decomposing broad competencies into more targeted sub-competencies, and consolidating overlapping elements while preserving their original conceptual coverage.

For example, the literature-derived competency *Ethical, Legal, and Societal Implications* was mapped simultaneously to Digital Citizenship and Ethical Competence and Impact of Computing on Society, reflecting its dual ethical–societal nature, and was subsequently refined into *AI Ethics*, *AI Safety and Risk Mitigation*, and *AI Societal Evaluation* within the AIEDF. This structured mapping-and-refinement process ensured that the resulting framework preserved conceptual completeness while improving pedagogical clarity and domain coherence.

The following table (Table 7) highlights how these seven core AI competencies align with the existing HDLF structure. By mapping specific skills and knowledge areas to relevant framework components, this table underscores the breadth of AI literacy necessary for comprehensive middle school education.

To maintain clarity and readability within the main text, only selected examples of cross-cutting AI competencies and their conceptual alignment with HDLF domains are presented here. The complete conceptual-fit mapping for all identified AI competencies is provided in the Appendix Table A1, while Table 8 introduces representative cases that illustrate the multi-domain alignment underpinning the AIEDF structure.

The AIEDF in Table 8 was developed by systematically aligning the seven AI competencies with the HDLF. This process involved decomposing broader competencies, such as splitting Ethical, Legal, and Societal Implications into more targeted areas like AI Ethics and AI Safety and Risk Mitigation to better align with sub-core areas (e.g., Ethics and Responsibility, Safety and Well-being). Additionally, competencies were renamed for clarity (for instance, Practical Application became Applied AI Development) and mapped to relevant core areas, such as computational concepts and digital citizenship. Overlapping themes were resolved through careful adjustments, embedding adaptability into lifelong learning and refining ethical, societal, and technical dimensions to address risks like bias and misinformation. The outcome is a structured framework that integrates AI literacy with broader digital competencies, emphasizing adaptability, interdisciplinary collaboration, and ethical responsibility for middle school education. Table 8 translates these mapped competencies into the final AIEDF, demonstrating how AI concepts, including ethics, data literacy, and creative applications, cohesively integrate with the HDLF domains Table 9.

**Table 9:** AI-enhanced educational digital framework (AIEDF): final integrated competency structure.

**Core area**	Sub core area	AI competency	**Origin (literature competency)**	**Focus**
**Core Computational Concepts and Skills**	Data Science and Analysis	AI Data Literacy	2. Data Literacy and Computational Thinking & 1. Fundamental AI Knowledge and Understanding	The ability to collect, analyze, interpret, and critically evaluate data used in AI systems. It encompasses an understanding of how data is generated, processed, and used by AI models, including awareness of biases, ethical considerations, and the impact of data-driven decision-making
	Programming and Algorithms	AI Algorithmic Design	*1. Fundamental AI Knowledge and Understanding &* *3. Practical Application & Operational Competence*	Building, fine-tuning, and optimizing AI models (e.g., neural networks, decision trees) for better performance and efficiency
	Computing Systems	Fundamental AI Knowledge and Understanding	*1. Fundamental AI Knowledge and Understanding*	Core AI concepts, including machine learning, neural networks, natural language processing, robotics, and the history and evolution of AI. Establishing a foundational understanding of AI principles, algorithmic logic, and real-world applications
**Digital Citizenship and Ethical Competence**	Ethics and Responsibility	AI Ethics	*7. Ethical, Legal, and Societal Implications &4. Critical Evaluation, Creativity, and Innovation*	Addressing ethical dilemmas (transparency, accountability, bias mitigation)
	Safety and Well-being	AI Safety and Risk Mitigation	*7. Ethical, Legal, and Societal Implications*	Managing AI risks (e.g., misinformation, **hallucination awareness**, cybersecurity, and mental health)
**Impact of Computing on Society**	Societal Impact	AI Societal Evaluation	*4. Critical Evaluation, Creativity, and Innovation & 7. Ethical, Legal, and Societal Implications*	Assessing AI’s societal risks/benefits (e.g., bias, automation, privacy)
	Equity and Inclusion	Inclusive AI Design	*7. Ethical, Legal, and Societal Implications*	Ensuring fairness, accessibility, and cultural sensitivity in AI systems
**Digital Competence and Lifelong Learning**	Problem Solving with Technology	Applied AI Development	*3. Practical Application & Operational Competence*	Hands-on coding, debugging, and deploying AI solutions
	Content Creation	AI-Driven Creativity	4. Critical Evaluation, Creativity, and Innovation	Leveraging AI tools for creative content generation by applying prompt literacy across text, images, music, and code
	Communication and Collaboration	Cross-Disciplinary AI Integration	5. Interdisciplinary Collaboration and Communication	Collaborating on AI projects with non-technical stakeholders

### Mapping AI competencies to bloom’s taxonomy

The rapid integration of AI into educational ecosystems necessitates a structured, evidence-based framework to align AI literacy with established pedagogical principles. Bloom’s Taxonomy, a hierarchical model categorizing cognitive skills into six levels (Remember, Understand, Apply, Analyze, Evaluate, Create)^81^, provides a robust scaffold for mapping AI competencies, ensuring clarity in learning outcomes and scientific rigor in skill progression. This mapping addresses a critical gap in AI education: the disconnect between technical proficiency (e.g., coding, data analysis) and higher-order cognitive skills (e.g., ethical reasoning, innovation)^28,82,83^.

In this study, Bloom alignment is used as a scaffold for sequenced learning expectations rather than a strict one-level classification. As AI competencies often involve multiple cognitive demands, they are represented as learning progressions across Bloom levels (e.g., Remember → understand → apply → analyze), reflecting how middle school learners typically develop competency over time. This progression-based interpretation reduces subjectivity by clarifying how competency can begin at lower cognitive demands and culminate in higher-order performance.

**Figure 2:**
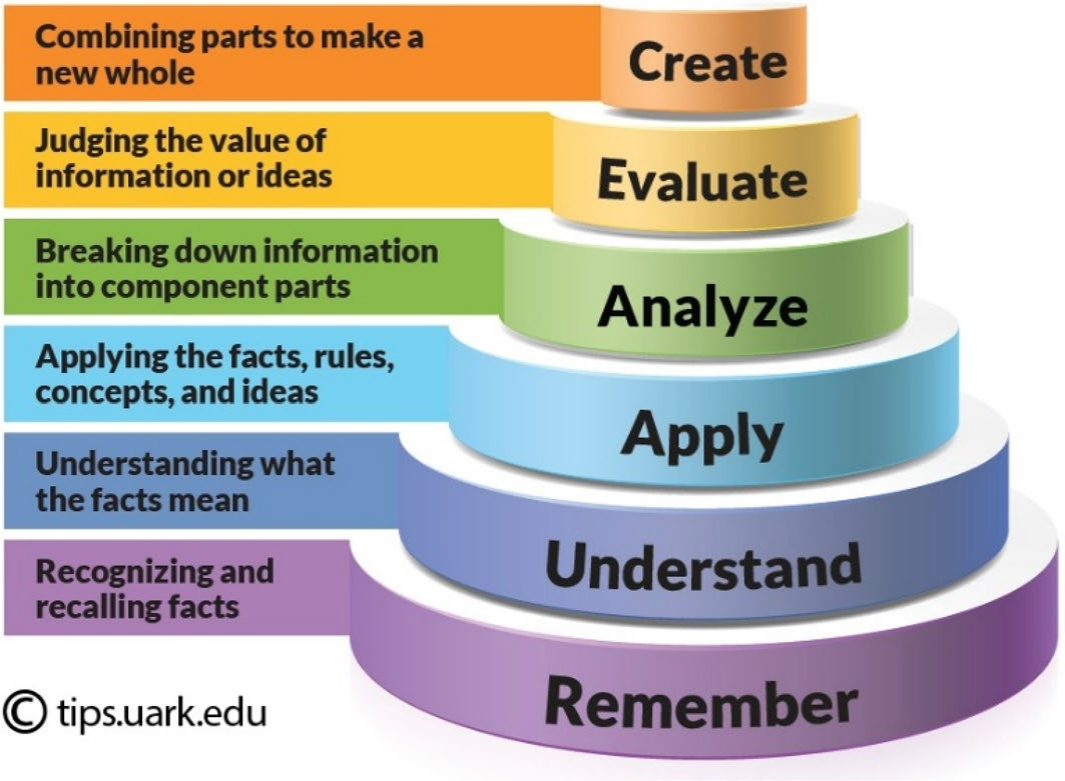
Bloom’s taxonomy resource:^86^.

Bloom’s Taxonomy (Fig. 2) provides the cognitive-process structure used to express AI competency learning progressions. The full AIEDF competency-to-Bloom learning progression mapping is reported in Appendix Table A2, while Table 10 provides selected Bloom-aligned learning objectives and assessment exemplars (Grades 6–8) to illustrate how these progressions can be operationalized in classroom practice. This process prioritizes completeness and logical flow. Foundational competencies, such as Fundamental AI Knowledge and Understanding, begin at the Remember/Understand levels to anchor learners in core computational concepts, while advanced competencies, such as Cross-Disciplinary AI Integration, extend toward Evaluate/Create to emphasize human-centric judgment and innovation. The framework adheres to curriculum design theory by maintaining consistency in terminology (e.g., computational thinking as a transversal layer that can span multiple levels) and by treating each competency as a trajectory that supports incremental cognitive growth^84,85^. Educators and policymakers can leverage this structured approach to design curricula that balance technical mastery with ethical accountability, fostering learners capable of harnessing AI’s potential while critically navigating its societal challenges.

**Table 10:** Selected bloom-aligned AI learning objectives and assessment exemplars for grades 6–8.

AI competency (AIEDF)	Bloom taxonomy learning progression	Concrete measurable learning outcome/objective (Bloom-aligned)	Assessment tasks aligned to Bloom (evidence)
AI Data Literacy	Understand → Apply → Analyze	(U) Interpret a provided dataset (table + chart) by correctly describing two patterns/trends. (A) Clean and organize a small dataset by applying rules for duplicates and missing values. (An) Identify one potential dataset bias/limitation and explain its likely effect on an AI outcome	(U) Dataset/chart interpretation short-response quiz (answer key). (A) Data-cleaning submission (before/after dataset + steps log). (An) Bias/limitation analysis note (template) scored with rubric (issue accuracy; impact explanation)
AI Algorithmic Design	Understand → Apply → Analyze → Evaluate	(U) Explain (with correct terms) training vs testing in supervised learning. (A) Build and run a basic classifier using a guided workflow and report test accuracy. (An) Compare two model runs and identify which change is likely to cause performance differences. (E) Justify the preferred model using at least two criteria (e.g., accuracy, error types, fairness)	(U) Concept check (short constructed response) scored with rubric. (A) Practical build log + results table (accuracy + example errors). (An) Comparative analysis worksheet scored with a checklist. (E) Written justification scored with a criteria-based rubric
Cross-Disciplinary AI Integration	Understand → Apply → Analyze → Create	(U) Explain an AI project goal to a non-technical audience using clear inputs/outputs and constraints. (A) Translate stakeholder needs into a simple specification (problem, users, data, success criteria). (An) Analyze trade-offs (accuracy vs interpretability; privacy vs utility) for the project context. (C) Produce a project plan/prototype proposal integrating domain needs and AI constraints	(U) Briefing (short text or 1–2 min) scored with rubric (clarity + correctness). (A) Requirements/spec document scored with checklist. (An) Trade-off analysis table scored with rubric. (C) Project plan/proposal artifact scored for feasibility and alignment to requirements

While the proposed framework maps AI competencies to Bloom’s Taxonomy, its practical relevance is strengthened by acknowledging well-documented limitations of large language models (LLMs). Because LLMs generate text by learning statistical patterns rather than grounded understanding, they can produce fluent but incorrect outputs (“hallucinations”) and may reproduce or amplify biases present in their training data, which makes human oversight essential in ethically sensitive classroom and societal contexts^87^.

Consistent with risk-governance guidance, educators should treat AI outputs as fallible artifacts requiring verification, context-aware interpretation, and accountability mechanisms, particularly when AI is used for evaluation-related or high-stakes judgments^87^. Cross-disciplinary collaboration between technical and societal domains is essential to align AI solutions with human needs, particularly in inclusive design for marginalized communities, which demands explicit integration of competencies such as Inclusive AI Design alongside AI Ethics. Furthermore, AI’s capacity to automate foundational tasks (e.g., adaptive quizzes) can liberate instructional time for higher-order learning activities, further supporting progression toward Analyze, Evaluate, and Create levels.

This Bloom’s Taxonomy alignment supports educators in sequencing AI learning from foundational to advanced cognitive demands. Rather than constraining each competency to a single Bloom level, the learning progression representation clarifies how competencies can be developed over time, guiding middle school learners toward increasingly complex AI tasks and critical thinking skills as they progress.

To reduce subjectivity, each LO was mapped using the revised Bloom cognitive-process verbs and assigned a progression only when the competency is intentionally revisited at increasing cognitive depth (Grades 6–8), with assessment evidence specified for each target level.

This Bloom’s Taxonomy mapping helps educators decide how deeply each AI competency should be taught. Some competencies are more foundational, requiring only Remember and Understand skills, while others invite learners to Apply, Analyze, Evaluate, or Create. This stratification ensures a developmentally appropriate approach, guiding middle school students toward increasingly complex AI tasks and critical thinking skills as they progress.

### Planned empirical validation

Although this study’s contribution is the systematic design and synthesis of the HDLF/AIEDF, we include an explicit validation pathway to support reproducibility and future evidence generation. The framework is intended to be validated through (i) structured expert review to evaluate content relevance, clarity, and curricular feasibility, followed by (ii) small-scale classroom pilots to assess implementation feasibility and preliminary learning/engagement outcomes, and (iii) subsequent larger-scale evaluations to test generalizability across contexts. To avoid overstating impact, all claims in the present manuscript are framed as design claims grounded in synthesis and alignment procedures rather than demonstrated classroom effectiveness.

## Limitations

The present study is conceptual in nature, focusing on the architectural synthesis of established digital literacy frameworks and the systematic mapping of AI competencies tailored for middle school education. As such, the primary limitation of this work is the absence of empirical validation. While AIEDF is grounded in developmental theory and international standards, it has not yet been pilot-tested in active middle school environments.

Furthermore, the framework’s practical usability for educators, who face specific challenges such as lack of preparedness and limited resources, has not been evaluated. Finally, because this research is non-experimental, it does not provide data regarding the framework’s direct impact on student learning outcomes or the long-term retention of AI literacy concepts. This study provides the necessary theoretical blueprint, while the real-world efficacy and implementation challenges remain subjects for future empirical inquiry.

Additionally, the reference set includes predominantly European- and English-language frameworks, which may introduce regional framing; therefore, cross-regional expert validation and localization studies are necessary to confirm transferability of the HDLF & AIEDF structure across diverse curricula.

Additionally, the reference set draws heavily on frameworks originating from Western institutional contexts. Consequently, certain competency areas, particularly digital citizenship and AI ethics, carry embedded cultural assumptions that may not translate straightforwardly to other regional contexts. We acknowledge that this framework is not universally applicable without qualification; researchers or practitioners seeking to apply the AIEDF in Asian educational systems or Global South contexts will need to undertake deliberate cultural localization. Therefore, cross-regional expert validation and cultural adaptation studies are essential to ensure the framework’s equitable, relevant, and effective implementation across diverse global curricula.

## Conclusion

This study addresses the critical need to equip middle school students with AI competencies by synthesizing four established digital education frameworks into a consolidated model and enriching it with AI-specific skills. The resulting framework integrates technical proficiency, ethical reasoning, and societal awareness, structured across four core domains: Core Computational Concepts and Skills, Digital Citizenship and Ethical Competence, Impact of Computing on Society, and Digital Competence and Lifelong Learning. The framework ensures a balanced focus on technical mastery and human-centric skills by mapping AI competencies such as algorithmic design, data literacy, ethical evaluation, and interdisciplinary collaboration to these domains.

Alignment with Bloom’s Taxonomy further strengthens its pedagogical rigor, guiding learners from foundational knowledge (Remember, Understand) to advanced application (Apply, Analyze) and critical innovation (Evaluate, Create). This progression empowers students to not only interact with AI tools but also assess their societal implications and contribute ethically to technological advancements. The framework’s emphasis on inclusiveness, safety, and adaptability prepares students to navigate an AI-driven future as informed creators and responsible digital citizens, bridging the gap between theoretical understanding and real-world problem-solving.

## Future work

Future work will empirically validate the AI-Enriched Digital Educational Framework through a staged evaluation plan. Phase 1 (expert validation): conduct a Delphi-style expert consensus study with curriculum specialists, AI education researchers, and experienced middle-school teachers to review each competency for relevance, clarity, developmental appropriateness, and feasibility; revise items iteratively until a stable consensus is reached. Phase 2 (pilot feasibility): implement a small pilot (e.g., 2–4 classes) using a short instructional sequence aligned to selected AIEDF competencies; collect feasibility indicators (teacher workload, usability, time-on-task, implementation fidelity) and preliminary student outcomes (pre/post knowledge checks, artifact-based rubrics, short engagement surveys). Phase 3 (effectiveness/generalizability): conduct a broader study across multiple schools/regions to test robustness and localization, comparing outcomes across different curricular contexts and teacher preparation levels. This staged approach is intended to provide evidence for both content validity (expert agreement) and practical validity (classroom feasibility and learning impact).

## Data Availability

This study is based on synthesizing and analyzing publicly available frameworks and published literature. No new primary datasets were generated or analyzed during the current study. The frameworks used (DigComp 2.2, K–12 Computer Science Framework, and Informatics Reference Frameworks) are publicly accessible via their official sources as cited in the References section. Further information can be requested from the corresponding author.

## See Tables 11 and 12

**Table 11:** shows the Full AI competency-to-HDLF competencies mapping (Grades 6–8).

**AI competency (literature-derived)**	**Mapped HDLF domain(s)**	**Conceptual fit**
1. Fundamental AI Knowledge and Understanding	Core Computational Concepts and Skills (Programming and Algorithms; Computing Systems; Data Science and Analysis)	This competency aligns with Core Computational Concepts and Skills because foundational AI literacy requires learners to recognize AI systems, understand core AI technologies (e.g., machine learning, natural language processing, robotics, and IoT), and interpret AI representations and decision processes. These capabilities depend on computational reasoning grounded in programming and algorithms, computing systems, and data-driven analysis, which collectively provide the conceptual basis necessary for understanding how AI systems function and support the development of more advanced AI skills.
2. Data Literacy and Computational Thinking	Core Computational Concepts and Skills (Data Science and Analysis)	This competency maps directly to Core Computational Concepts and Skills, particularly Data Science and Analysis, because AI systems are fundamentally driven by data. Learners a, must therefore develop abilities in data collection, cleaning, analysis, visualization, and interpretation, alongside algorithmic reasoning and coding practices that enable understanding of how AI models learn from data and generate outputs. These knowledge elements are conceptually contained within the computational domain and do not intrinsically extend to ethical or societal HDLF domains.
3. Practical Application and Operational Competence	Core Computational Concepts and Skills; Digital Competence and Lifelong Learning	This competency aligns with both Core Computational Concepts and Skills and Digital Competence and Lifelong Learning. The development and use of AI applications require computational abilities such as programming, debugging, model interaction, and algorithmic problem solving, which belong to the computational domain. Simultaneously, applying AI in real-world contexts involves iterative experimentation, project-based implementation, and adaptive problem solving, which correspond to the lifelong learning and practical engagement dimensions of the HDLF.
4. Critical Evaluation, Creativity, and Innovation	Impact of Computing on Society; Digital Competence and Lifelong Learning; Digital Citizenship and Ethical Competence	This competency aligns with Impact of Computing on Society, Digital Competence and Lifelong Learning, and Digital Citizenship and Ethical Competence because critically evaluating AI outputs requires understanding their societal consequences, potential biases, and limitations. Creative and innovative uses of AI involve problem solving, content generation, and exploratory design practices that correspond to lifelong learning and technological application. At the same time, responsible and ethical innovation requires judgment regarding fairness, transparency, and appropriate AI use, linking the competency to ethical and citizenship-oriented dimensions of the HDLF.
5. Interdisciplinary Collaboration and Communication	Digital Competence and Lifelong Learning; Impact of Computing on Society	This competency maps to Digital Competence and Lifelong Learning and Impact of Computing on Society because effective AI integration depends on the ability to communicate technical concepts clearly, collaborate across disciplinary boundaries, and apply AI knowledge within diverse real-world contexts. These practices reflect both collaborative digital engagement and the societal application of computing, positioning interdisciplinary teamwork as a bridge between technological understanding and socially meaningful AI use.
6. Adaptability and Lifelong Learning	Digital Competence and Lifelong Learning	This competency aligns directly with Digital Competence and Lifelong Learning because the rapid evolution of AI technologies requires continuous skill development, self-directed learning, and the capacity to adapt to emerging tools and knowledge. These characteristics correspond conceptually to lifelong learning within the HDLF and do not inherently require mapping to computational, ethical, or societal domains beyond their role in sustaining long-term technological competence.
7. Ethical, Legal, and Societal Implications	Digital Citizenship and Ethical Competence; Impact of Computing on Society	This competency aligns with Digital Citizenship and Ethical Competence and Impact of Computing on Society because it encompasses both individual ethical responsibility in AI use—such as privacy, fairness, transparency, and accountability—and broader societal consequences, including bias, misinformation, security risks, and equity considerations. The dual focus on personal ethical conduct and collective societal impact necessitates mapping across both HDLF domains to fully represent the scope of ethical and socio-technical understanding required for responsible AI engagement.

**Table 12:** Shows the full AIEDF competency-to-bloom learning progression mapping (Grades 6–8).

**AI competency (AIEDF)**	**Bloom taxonomy learning progression**	**Concrete measurable learning outcome/objective (Bloom-aligned)**	**Assessment tasks aligned to Bloom (evidence)**
AI Data Literacy	Understand → Apply → Analyze	(U) Interpret a provided dataset (table + chart) by correctly describing two patterns/trends. (A) Clean and organize a small dataset by applying the given rules for duplicates and missing values. (An) Identify one potential dataset bias/limitation and explain its likely effect on an AI outcome.	(U) Dataset/chart interpretation short-response quiz (answer key). (A) Data-cleaning submission (before/after dataset + steps log). (An) Bias/limitation analysis note (template) scored with rubric (issue accuracy; impact explanation).
AI Algorithmic Design	Understand → Apply → Analyze → Evaluate	(U) Explain (with correct terms) training vs testing in supervised learning. (A) Build and run a basic classifier using a guided workflow and report test accuracy. (An) Compare two model runs and identify which change is likely to cause performance differences. (E) Justify the preferred model using at least two criteria (e.g., accuracy, error types, fairness).	(U) Concept check (short constructed response) scored with rubric. (A) Practical build log + results table (accuracy + example errors). (An) Comparative analysis worksheet scored with a checklist. (E) Written justification scored with a criteria-based rubric.
Fundamental AI Knowledge and Understanding	Remember → Understand → Apply	(R) Define core AI terms (AI, model, dataset, training, inference). (U) Classify everyday examples as AI vs non-AI and explain the reason in one sentence each. (A) Given a scenario, describe the system’s input → process → output at a high level.	(R) Terminology quiz (matching/definitions). (U) Scenario classification task scored with rubric (label + explanation). (A) Input/process/output mapping worksheet scored with a checklist.
AI Ethics	Understand → Analyze → Evaluate → Create	(U) Explain one AI-ethics issue (e.g., transparency, accountability, bias) using one relevant example. (An) Identify stakeholders and potential harms/benefits in a given AI use case. (E) Judge acceptability using explicit criteria (fairness, privacy, accountability) and justify. (C) Propose one concrete mitigation and explain how it addresses the issue.	(U) Short response scored with rubric (concept accuracy + example relevance). (An) Case-analysis sheet (stakeholders + harms/benefits) scored with checklist. (E) Criteria-based argument scored with a rubric. (C) Mitigation proposal (one-page) scored for feasibility + alignment.
AI Safety and Risk Mitigation	Understand → Apply → Analyze → Evaluate	(U) Describe two AI-related risks (e.g., misinformation, AI hallucinations, privacy, cybersecurity) in clear terms. (A) Apply a safety checklist to an AI tool/use-case and mark compliant vs non-compliant items. (An) Explain how a specific risk could occur (cause → pathway → outcome) in a scenario. (E) Select the best mitigation among options and justify using severity and likelihood.	(U) Scenario-based risk identification quiz. (A) Completed safety checklist artifact. (An) Cause–pathway diagram scored with rubric. (E) Mitigation choice justification scored with rubric (severity/likelihood reasoning).
AI Societal Evaluation	Understand → Analyze → Evaluate	(U) Summarize one positive and one negative societal impact of an AI application. (An) Analyze how bias or automation could affect different groups in a given context. (E) Evaluate an AI application using an impact rubric (benefits, harms, fairness, privacy) and justify the rating.	(U) Structured summary scored with rubric. (An) Group-impact analysis worksheet scored with checklist. (E) Completed impact rubric + justification paragraph scored with rubric.
Inclusive AI Design	Understand → Apply → Analyze → Create	(U) Explain what fairness/accessibility means in an AI context using one example. (A) Apply inclusive design checks (accessibility/representation) to a scenario and identify missing elements. (An) Analyze how one AI design choice may disadvantage a specific group. (C) Redesign one aspect (data, interface, or decision rule) to improve inclusion and explain the expected improvement.	(U) Short response scored with rubric. (A) Inclusive checklist completion artifact. (An) Impact-on-group analysis paragraph scored with rubric. (C) Redesign submission (annotated sketch/spec + rationale) scored for alignment to inclusion goal.
Applied AI Development	Apply → Analyze → Create	(A) Implement a guided AI workflow (collect data → train → test) and run it end-to-end. (An) Debug an underperforming model by identifying at least one likely cause from results/logs. (C) Build a small AI prototype for a defined problem and demonstrate it with test cases.	(A) Lab submission (workflow steps + outputs). (An) Debug report (issue, evidence, fix, result) scored with rubric. (C) Prototype demo + test-case evidence (inputs/outputs) scored with performance rubric.
AI-Driven Creativity	Understand → Apply → Create → Evaluate	(U) Explain what a generative AI tool does and identify one limitation (e.g., errors, bias). (A) Generate a draft artifact (text/image/code) following given constraints. (C) Produce a final artifact by demonstrating prompt literacy (iterating prompts/edits) and documenting at least two improvement iterations. (E) Critique the final artifact using a rubric (quality, originality, correctness, ethics) and justify the score.	(U) Short concept check scored with rubric. (A) Draft artifact + prompt log. (C) Iteration portfolio (versions + changes) scored with rubric. (E) Self/peer evaluation rubric + justification paragraph.
Cross-Disciplinary AI Integration	Understand → Apply → Analyze → Create	(U) Explain an AI project goal to a non-technical audience using clear inputs/outputs and constraints. (A) Translate stakeholder needs into a simple specification (problem, users, data, success criteria). (An) Analyze trade-offs (accuracy vs interpretability; privacy vs utility) for the project context. (C) Produce a project plan/prototype proposal integrating domain needs and AI constraints.	(U) Briefing (short text or 1–2 min) scored with rubric (clarity + correctness). (A) Requirements/spec document scored with checklist. (An) Trade-off analysis table scored with rubric. (C) Project plan/proposal artifact scored for feasibility and alignment to requirements.
